# Assessment of Periodontal Health Status and Treatment Needs Among Pregnant Women

**DOI:** 10.7759/cureus.65267

**Published:** 2024-07-24

**Authors:** Sana Zahra, Hira Asghar, Nousheen Khan, Mehmood Ahmed Rana, Shamsher Ali, Rabia Asad, Hafiz Muhammad Abu Bakar Siddique

**Affiliations:** 1 Department of Prosthodontics, Multan Medical and Dental College, Multan, PAK; 2 Science of Dental Materials, Azra Naheed Dental College, Lahore, PAK; 3 Department of Periodontology, Multan Medical and Dental College, Multan, PAK; 4 Department of Operative Dentistry and Endodontics, Multan Medical and Dental College, Multan, PAK; 5 Department of Dental Materials, Akhtar Saeed Medical and Dental College, Lahore, PAK; 6 Department of Oral Biology, Multan Medical and Dental College, Multan, PAK

**Keywords:** birth outcomes, oral hygiene, treatment needs, pregnant women, periodontal health

## Abstract

Background

Pregnancy-related periodontal health is vital for maternal and fetal well-being, with implications on birth outcomes. However, comprehensive data on periodontal health among pregnant women in Pakistan are lacking. This research aimed to assess the periodontal health status and treatment needs among pregnant women in Pakistan.

Methodology

This study conducted at Multan Medical and Dental College in Multan, Pakistan, utilized a cross-sectional design over one year from January 2023 to December 2023. It enrolled 230 pregnant women from the prenatal care clinic, employing strict inclusion and exclusion criteria to ensure data integrity. Data collection involved a systematic questionnaire and clinical examination by qualified dental practitioners, covering demographic details, obstetric history, dental hygiene habits, and periodontal health parameters. Statistical analysis included descriptive statistics, chi-square tests, and logistic regression to evaluate periodontal health status determinants.

Results

This study, comprising 230 participants, delineates key demographic and periodontal health indicators. Notably, the age group between 26 and 30 years made up the greatest percentage (n = 87, 37.83%), followed by the age group between 18 and 25 years (n = 58, 25.22%). The chi-square test showed a significant association between age and periodontal health (χ² = 8.23, df = 3, p = 0.041). University-educated participants showed decreased periodontal risks (odds ratio = 0.51, p = 0.037), with education level also emerging as a significant factor (χ² = 12.76, df = 2, p = 0.002). Regarding dental hygiene, 44.35% of people brushed twice a day, and 27.83% flossed every day. Periodontal data revealed that 53.04% of individuals had gingivitis and that the mean probing depth was 3.22 mm. Scaling and root planing were the most requested therapy (50.00%).

Conclusions

This study provides valuable insights into the periodontal health status and treatment needs of pregnant women in Pakistan. Investigative analyses including chi-square tests and logistic regression identified significant associations between demographic factors, oral hygiene practices, and periodontal health outcomes among pregnant women in Pakistan, emphasizing the need for tailored interventions to enhance maternal and child health.

## Introduction

Pregnancy-related periodontal health is crucial for both the growing baby and the mother [1,2]. There seems to be a reciprocal link between periodontal illnesses and unfavorable pregnancy outcomes, such as low birth weight and premature delivery [3,4]. Although this association is becoming more well-acknowledged, there is still a lot we do not know about Pakistani pregnant women’s periodontal health and treatment requirements [5].

Like in many other developing nations, maternal and child health continues to be a major public health problem in Pakistan [6,7]. Given the widespread use of inadequate oral hygiene habits and restricted access to dental care services, the burden of periodontal diseases among expectant mothers is likely significant [8]. However, there is a dearth of thorough information about Pakistani pregnant women’s periodontal health, which makes it difficult to establish focused treatments and legislative measures [9].

Moreover, the frequency and severity of pregnancy-related periodontal diseases can vary based on cultural practices, dietary preferences, and access to oral health education and services across different geographic and socioeconomic settings [10,11]. Designing successful treatments that are suited to the unique requirements of this vulnerable group requires an understanding of these contextual elements [12].

Furthermore, the majority of the literature concentrates on high-income nations, with little emphasis on low- and middle-income nations like Pakistan. The results of studies done in high-income environments may not apply directly to the situation in Pakistan because of the differences in healthcare resources and infrastructure. Therefore, to support evidence-based treatments and the development of policies targeted at enhancing the periodontal health of pregnant women in Pakistan, there is an urgent need for locally relevant research.

It is essential to close this research gap if Pakistan is to see improvements in the health of mothers and children. This study intends to close this important information gap and aid in the creation of focused interventions to enhance mother and child health outcomes in Pakistan by thoroughly evaluating the periodontal health status and treatment requirements of pregnant women. Therefore, the objective of this research was to assess the periodontal health status and treatment needs of pregnant women in Pakistan.

## Materials and methods

Study design and settings

This study was conducted in Multan Medical and Dental College, Multan, Pakistan, using a cross-sectional design from January 2023 to December 2023.

Inclusion and exclusion criteria

The study included female participants who were at least 18 years old and were registered in the prenatal care clinic at Multan Medical and Dental College. The study excluded pregnant patients receiving treatment for periodontal disorders, those with a history of chronic medical illnesses (such as diabetes mellitus and hypertension), and those who declined to participate.

Sample size

In this study, 230 pregnant women were enrolled with a 95% confidence level and a 5% margin of error, according to the predicted frequency of periodontal disorders among expectant mothers in comparable circumstances.

Data collection

A systematic questionnaire and a clinical examination were used to gather information on the state of periodontal health and the need for treatment. Demographic data, obstetric history, dental hygiene habits, and periodontal health awareness were included in the questionnaire. Periodontal characteristics including probing depth and clinical attachment loss were typically measured using UNC-15 probes, while gingival inflammation and calculus presence were assessed visually by the dental practitioners and detected by tactile examination, meaning they were examined and judged based on their appearance rather than measured with instruments. Qualified dental practitioners collected all information following accepted practices.

Variables

In the study, variables included age (categorized into 18-25, 26-30, 31-35, and >35 years), education level (school, college, and university), occupation (housewife, employed, and unemployed), and income level (low, middle, and high). Periodontal health parameters measured included probing depth (depth of the periodontal pocket in mm), clinical attachment loss (distance from the cementoenamel junction to the bottom of the pocket in mm), gingival index (severity of gingival inflammation), plaque index (amount of plaque), calculus index (amount of calculus), bleeding on probing (percentage of sites bleeding upon probing), tooth mobility (movement of teeth in mm), furcation involvement (extent of disease between tooth roots in mm), gingival recession (distance from the cementoenamel junction to the gum margin in mm), and periodontal pocket depth (average depth of pockets in mm). Oral hygiene habits included the frequency of tooth brushing (fewer than once a day, once a day, twice a day, more than twice a day), flossing (never, rarely, sometimes, daily), and mouthwash use (yes/no).

Statistical analysis

The study participants’ periodontal parameters and demographics were compiled using descriptive statistics. The chi-square test and logistic regression analysis are two examples of inferential statistics that were used to investigate relationships between different clinical and demographic factors and the state of periodontal health. Statistical significance was established at p-values <0.05.

Ethical approval

The Institutional Review Board of Multan Medical and Dental College in Multan, Pakistan, granted ethical approval for this research, as documented in Letter No. C-37-928, dated November 22, 2021. Before the commencement of the study, all participants provided written informed consent, and data confidentiality was strictly maintained throughout the research.

## Results

The demographic details of the study participants (n = 230) are shown in Table 1. In total, 87 (37.83%) patients were in the 26-30-year age group, with the greatest percentage of participation across the age categories, followed by 58 (25.22%) patients in the 18-25-year age group, 53 (23.04%) patients in the 31-35-year age group, and 32 (13.91%) patients in the over 35-year age group. Most participants had completed different levels of education, with 68 (29.57%) patients having finished schooling, 74 (32.17%) patients having achieved a university degree, and 88 (38.26%) patients having completed their college education. Regarding occupation, 121 (52.61%) patients were housewives, followed by 66 (28.70%) patients who were working and 43 (18.70%) unemployed patients. With 106 (46.09%) patients, the middle-income group accounted for the biggest percentage in terms of income level. The low-income group had 73 (31.74%) patients, and the high-income group had 51 (22.17%) patients.

**Table 1:** Demographic characteristics of study participants (n = 230).

Variable		Number of patients (n)	Percentage (%)
Age (years)	18–25	58	25.22
	26–30	87	37.83
	31–35	53	23.04
	>35	32	13.91
Education level	School	68	29.57
	College	88	38.26
	University	74	32.17
Occupation	Housewife	121	52.61
	Employed	66	28.70
	Unemployed	43	18.70
Income level	Low	73	31.74
	Middle	106	46.09
	High	51	22.17

The obstetric history of the research participants (n = 230) is shown in Table 2. Regarding gravidity, 147 (63.91%) patients were multigravida, and 83 (36.09%) patients were primigravida. There were also other participants in the study. Regarding parity, 96 (41.74%) patients were nulliparous, and 134 (58.26%) multiparous patients were included in the research. Overall, 92 (40.00%) patients were in the second trimester, 71 (30.87%) patients were in the third trimester, and 67 (29.13%) patients were in the first trimester, according to the gestational age distribution.

**Table 2:** Obstetric history of study participants (n = 230).

Variable		Number of patients (n)	Percentage (%)
Gravidity	Primigravida	83	36.09
	Multigravida	147	63.91
Parity	Nulliparous	96	41.74
	Multiparous	134	58.26
Gestational age	First trimester	67	29.13
	Second trimester	92	40.00
	Third trimester	71	30.87

The oral hygiene routines of the 230 research participants are summarized in Table 3. Regarding the frequency of tooth brushing, 102 (44.35%) patients brushed twice a day, 41 (17.82%) more than twice a day, 62 (26.96%) once a day, and 25 (10.87%) less than once a day. Regarding the frequency of flossing, 64 (27.83%) patients reported flossing daily, followed by 77 (33.47%) who flossed infrequently, 46 (20.00%) who flossed sometimes, and 43 (18.70%) who flossed never. In terms of mouthwash use, 110 (47.83%) patients did not use it, while 120 (52.17%) reported using it.

**Table 3:** Oral hygiene practices of study participants (n = 230).

Variable		Number of patients (n)	Percentage (%)
Frequency of tooth brushing	Fewer than once a day	25	10.87
	Once a day	62	26.96
	Twice a day	102	44.35
	More than twice a day	41	17.82
Frequency of flossing	Rarely	77	33.47
	Occasionally	46	20.00
	Daily	64	27.83
	Never	43	18.70
Mouthwash usage	Yes	120	52.17
	No	110	47.83

The periodontal parameters of the 230 research participants are summarized in Table 4. The mean probing depth was 3.22 mm, and the standard deviation (SD) was ±0.78 mm. The mean clinical attachment loss was 2.45 mm (±0.61 mm). The mean calculus index was 2.10 (±0.58), the mean gingival index was 1.78 (±0.45), and the mean plaque index was 1.92 (±0.52). Upon probing, 25.43% (±7.12%) of bleeding was found. The mean measurement of tooth mobility was 0.34 mm (±0.12 mm), while the mean measurement of furcation involvement was 0.17 mm (±0.09 mm). The gingival recession was 1.10 mm (±0.36 mm) on average. Ultimately, the average depth of the periodontal pocket was 4.65 mm (±1.23 mm).

**Table 4:** Periodontal parameters of study participants (n = 230).

Parameter	Mean ± SD
Probing depth (mm)	3.22 ± 0.78
Clinical attachment loss (mm)	2.45 ± 0.61
Gingival index	1.78 ± 0.45
Plaque index	1.92 ± 0.52
Calculus index	2.10 ± 0.58
Bleeding on probing (%)	25.43 ± 7.12
Tooth mobility (mm)	0.34 ± 0.12
Furcation involvement (mm)	0.17 ± 0.09
Gingival recession (mm)	1.10 ± 0.36
Periodontal pocket depth (mm)	4.65 ± 1.23

**Figure 1:**
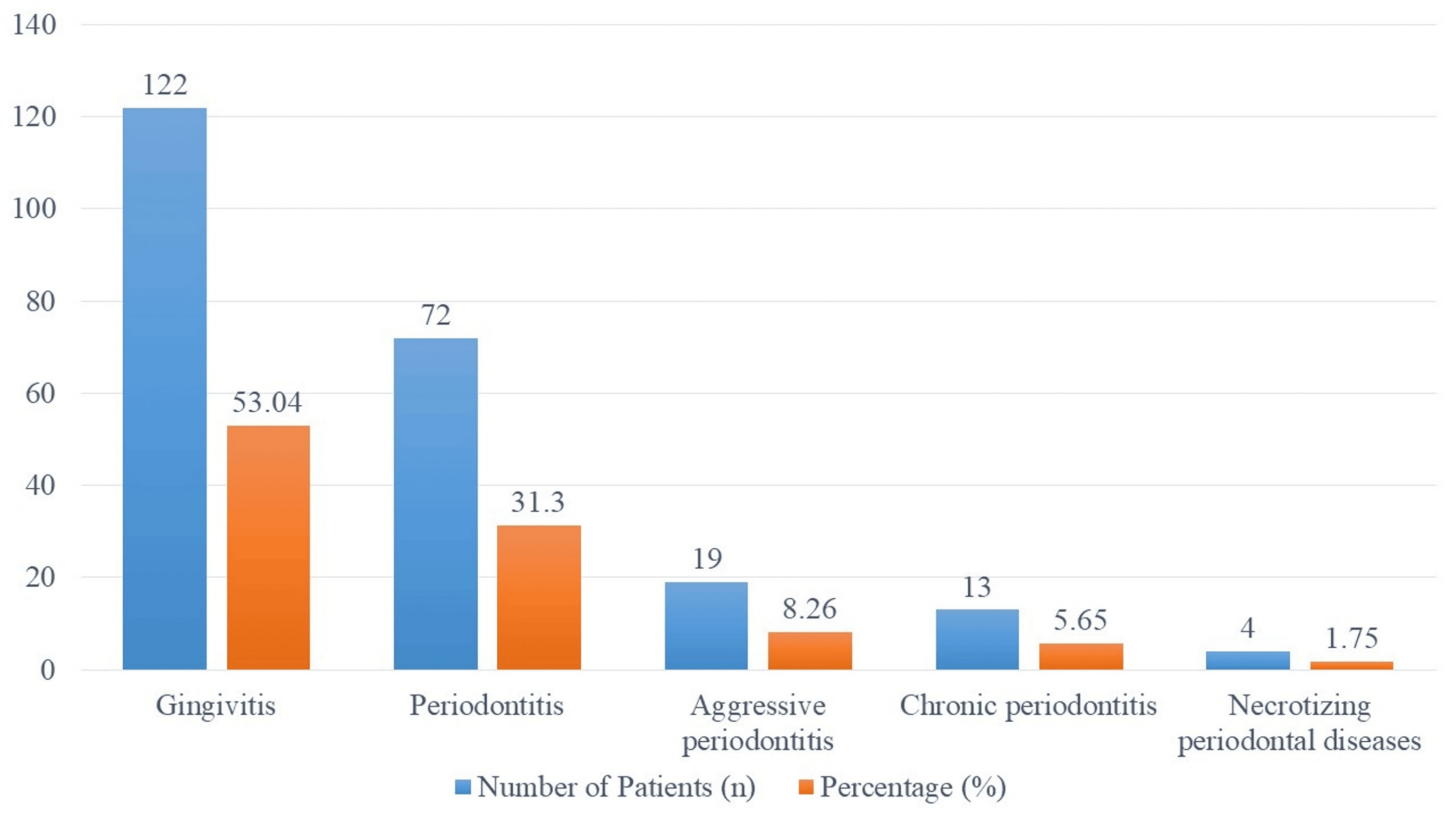
Prevalence of periodontal diseases among study participants (n = 230).

The frequency of periodontal disorders among the 230 research participants is shown in Figure 1. Gingivitis was the most common ailment, affecting 122 individuals or 53.04% of the sample. The second most prevalent condition, involving 72 (31.30%) individuals, was periodontitis. Of the patients, 19 (8.26%) had aggressive periodontitis. Overall, 13 (5.65%) patients had chronic periodontitis. Lastly, four (1.75%) patients had necrotizing periodontal infections.

The findings of the chi-square analysis, which examined the relationships between several variables and the periodontal health status of the research participants (n = 230), are shown in Table 5. For every variable, the degrees of freedom (df), p-values, and chi-square values are provided. Age (χ² = 8.23, df = 3, p = 0.041), education level (χ² = 12.76, df = 2, p = 0.002), employment (χ² = 6.45, df = 2, p = 0.040), and gestational age (χ² = 4.12, df = 2, p = 0.034) showed statistically significant associations with periodontal health status. Furthermore, there was a significant association between the periodontal health status and the frequency of teeth brushing (χ² = 9.87, df = 3, p = 0.021) and flossing (χ² = 7.55, df = 3, p = 0.025). While they did not approach statistical significance, the following variables exhibited tendencies toward significance: parity (χ² = 2.98, df = 1, p = 0.084), income level (χ² = 5.91, df = 2, p = 0.055), gravidity (χ² = 3.67, df = 1, p = 0.055), and mouthwash use (χ² = 5.33, df = 1, p = 0.070).

**Table 5:** Chi-square analysis of factors associated with periodontal health status among study participants (n = 230).

Variable	Chi-square value	Degrees of freedom	P-value
Age	8.23	3	0.041
Education level	12.76	2	0.002
Occupation	6.45	2	0.040
Income level	5.91	2	0.052
Gravidity	3.67	1	0.055
Parity	2.98	1	0.084
Gestational age	4.12	2	0.034
Frequency of tooth brushing	9.87	3	0.021
Frequency of flossing	7.55	3	0.025
Mouthwash usage	5.33	1	0.070

The findings of the logistic regression analysis, which evaluated the variables predicting the periodontal health status of the research participants (n = 230), are shown in Table 6. For every variable, odds ratios (ORs) are given together with their 95% confidence intervals and p-values. Considerable correlations were noted for certain age groups, with older age groups showing larger ORs than the reference group (18-25 years old). In particular, those between the ages of 31 and 35 years (OR = 2.45, p = 0.016) and above 35 years (OR = 3.12, p = 0.009) showed significantly higher chances of having periodontal problems than those between the ages of 18 and 25 years. Significant correlations were also found between education level and periodontal problems. Those with a university degree had a decreased chance of developing periodontal problems than those with just a high school education (OR = 0.51, p = 0.037). In this study, no statistically significant relationships were found between periodontal health status and other factors, such as employment and income level. Furthermore, there were no noteworthy correlations between the periodontal health status and gestational age in the second or third trimesters compared to the first trimester.

**Table 6:** Logistic regression analysis of factors predicting periodontal health status among study participants (n = 230).

Variable	Odds ratio	95% confidence interval	P-value
Age (26–30 vs. 18–25)	1.78	0.92–3.44	0.087
Age (31–35 vs. 18–25)	2.45	1.18–5.09	0.016
Age (>35 vs. 18–25)	3.12	1.32–7.35	0.009
Education level (college vs. school)	0.72	0.39–1.32	0.284
Education level (university vs. school)	0.51	0.27–0.96	0.037
Occupation (employed vs. housewife)	0.84	0.46–1.52	0.567
Occupation (unemployed vs. housewife)	1.15	0.58–2.29	0.692
Income Level (middle vs. low)	0.93	0.51–1.70	0.822
Income Level (high vs. low)	1.32	0.63–2.75	0.457
Gestational age (second trimester vs. first trimester)	0.91	0.47–1.77	0.788
Gestational age (third trimester vs. first trimester)	1.26	0.64–2.48	0.509

**Figure 2:**
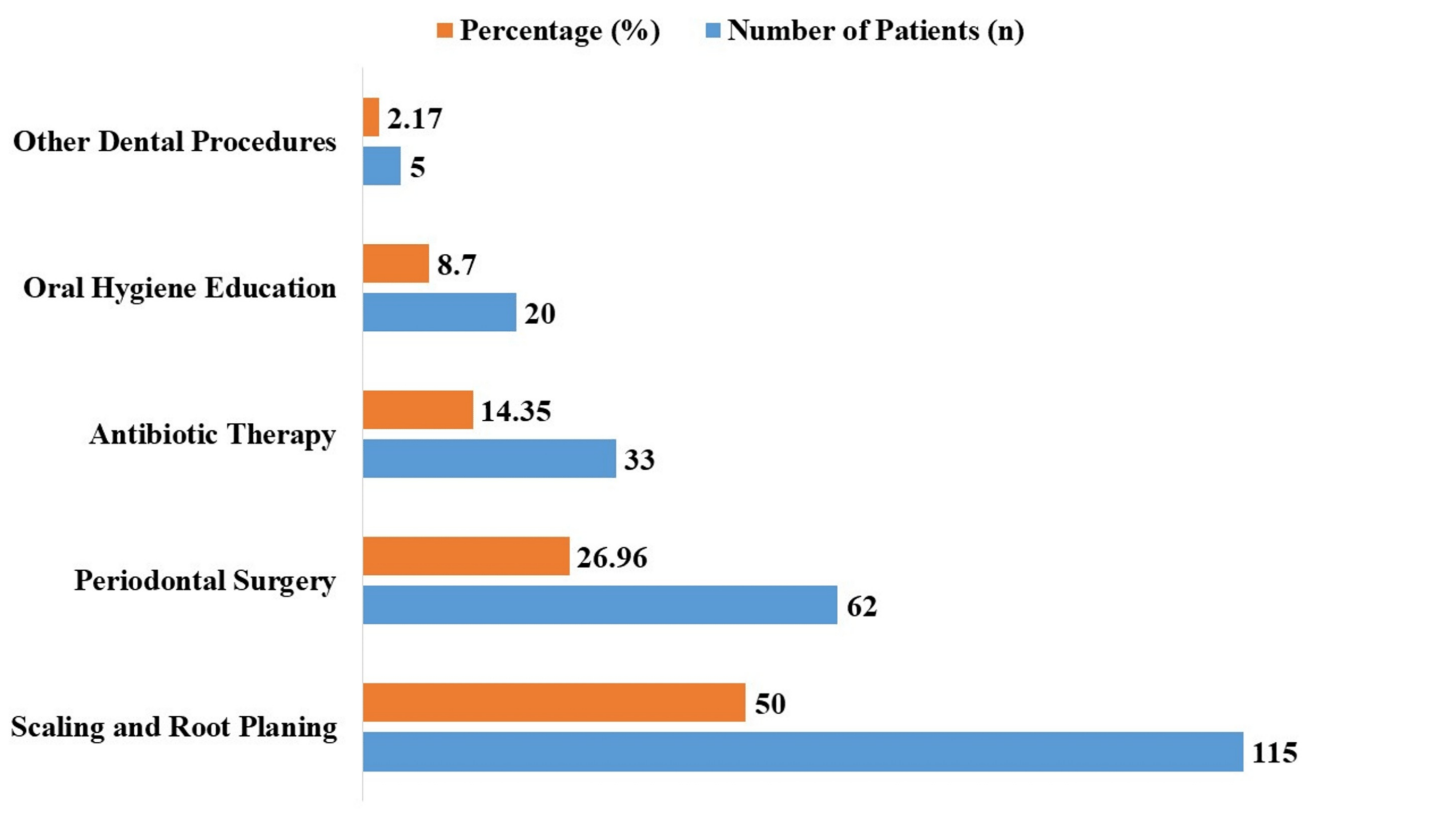
Treatment needs among study participants (n = 230).

The research participants’ (n = 230) treatment demands are shown in Figure 2. Of the treatments needed, scaling and root planing accounted for the majority, being needed in 115 (50.00%) patients. Overall, 33 (14.35%) patients had antibiotic treatment after 62 (26.96%) patients had periodontal surgery. Furthermore, five (2.17%) patients required further dental treatments, and 20 (8.70%) patients needed instruction on oral hygiene.

## Discussion

The present study investigates the periodontal health status and treatment needs among pregnant women in Pakistan, addressing a critical yet under-researched area that impacts maternal and fetal health outcomes. The findings reveal pertinent demographic characteristics and periodontal health indicators among the 230 participants. A notable observation is that individuals aged 26-30 years constituted the largest group (37.83%), with age demonstrating a statistically significant association with periodontal health (χ² = 8.23, df = 3, p = 0.041). This aligns with a previous study emphasizing age as a crucial factor influencing periodontal status during pregnancy [13].

Moreover, education level emerged as a significant determinant, with university-educated participants exhibiting reduced periodontal risks (OR = 0.51, p = 0.037) compared to those with lower educational attainment. This finding resonates with international research indicating that higher educational attainment correlates with better periodontal health outcomes across various countries [14,15]. The prevalence of gingivitis among the study participants in our research, affecting 53.04% of individuals, aligns with findings from previous studies that also highlight high rates of gingivitis during pregnancy [16]. Additionally, the mean probing depth of 3.22 mm observed in our study corresponds to similar measurements reported in research focusing on periodontal health in pregnant populations [17]. These consistent findings underscore the commonality of periodontal issues among pregnant women and emphasize the need for targeted interventions to mitigate these risks.

The logistic regression analysis further elucidates factors influencing periodontal health status, with older age groups (31-35 years and >35 years) demonstrating significantly higher odds of periodontal problems compared to younger age groups (OR = 2.45, p = 0.016 for 31-35 years; OR = 3.12, p = 0.009 for >35 years). This finding is consistent with other findings indicating that hormonal changes and age-related physiological alterations during pregnancy can exacerbate periodontal conditions [18,19]. Understanding these risk factors is essential for developing targeted preventive strategies and promoting maternal oral health during pregnancy.

Furthermore, the study identifies oral hygiene behaviors as crucial determinants of periodontal health, with significant associations found between the frequency of tooth brushing and flossing and periodontal status (χ² = 9.87, df = 3, p = 0.021 and χ² = 7.55, df = 3, p = 0.025, respectively). These results align with previous research studies highlighting the impact of oral hygiene practices on periodontal health outcomes among pregnant women [20,21]. Effective oral health education tailored to cultural norms and practices could potentially enhance these behaviors and subsequently improve periodontal outcomes. This underscores the need for targeted interventions aimed at promoting regular oral hygiene practices to mitigate periodontal disease risks during pregnancy.

The study conducted at Multan Medical and Dental College, Pakistan, employed a cross-sectional design over a one-year period to investigate periodontal health among pregnant women in prenatal care. Robust data collection methods, including systematic questionnaires and clinical examinations by qualified practitioners, provided comprehensive insights into periodontal parameters and associated factors. However, limitations of the current study include the reliance on self-reported data and clinical assessment for certain parameters, which may introduce biases. The study’s exclusion criteria might limit the generalizability of findings to pregnant women actively seeking prenatal care. Looking forward, future research could benefit from longitudinal designs to track changes in periodontal health across different trimesters of pregnancy. Incorporating advanced diagnostic tools and biomarkers could enhance the accuracy of assessments, potentially leading to more targeted preventive strategies and interventions to address current limitations and contribute to improving periodontal health management for pregnant women globally.

## Conclusions

This study provides insights into the important but little-researched topic of periodontal health in Pakistani pregnant women. The results highlight the strong correlations that exist between the state of periodontal health and several behavioral and demographic characteristics, including age, education level, and oral hygiene habits. The evaluation of treatment requirements also emphasizes the wide range of therapies needed to treat periodontal diseases in this group. This study offers insightful information that may guide focused actions and policies meant to enhance the health of Pakistani women and their children.

## Appendices

Questionnaire

Participant Information

Participant name (optional):

Date of birth:

Age:

Gender:

Male

Female

Demographic Information

Education level:

School

College

University

Occupation:

Housewife

Employed (specify job title: ____________)

Unemployed

Monthly income level:

Low

Middle

High

Obstetric History

Gravidity:

Primigravida (first pregnancy)

Multigravida (subsequent pregnancies)

Parity:

Nulliparous (no prior births)

Multiparous (one or more prior births)

Gestational age (current pregnancy):

First trimester

Second trimester

Third trimester

Oral Hygiene Practices

Frequency of tooth brushing per day:

Less than once a day

Once a day

Twice a day

More than twice a day

Frequency of flossing:

Never

Occasionally

Once a day

Daily

Use of mouthwash:

Yes

No

Periodontal Health Status

Have you been diagnosed with any periodontal disease?

Yes (Specify type: ____________)

No

Have you received any periodontal treatment during your current pregnancy?

Yes (Specify treatment: ____________)

No

Are you currently experiencing any symptoms related to your periodontal health (e.g., bleeding gums, tooth mobility)?

Yes

No

Treatment Needs

Based on your current periodontal health status, which of the following treatments have you been advised to undergo?

Scaling and root planing

Antibiotic treatment

Periodontal surgery

Other dental treatments (Specify: ____________)

Instruction on oral hygiene

If applicable, please specify any additional treatments recommended by your healthcare provider:
